# *In vivo* 3D photoacoustic and ultrasound analysis of hypopigmented skin lesions: A pilot study

**DOI:** 10.1016/j.pacs.2025.100705

**Published:** 2025-03-08

**Authors:** Minseong Kim, Ju Hee Han, Junho Ahn, Esther Kim, Chul Hwan Bang, Chulhong Kim, Ji Hyun Lee, Wonseok Choi

**Affiliations:** a Department of Convergence IT Engineering, Electrical Engineering, Mechanical Engineering, and Medical Science and Engineering, POSTECH-CATHOLIC Biomedical Engineering Institute, Medical Device Innovation Center, Pohang University of Science and Technology, Pohang, Republic of Korea; b Department of Dermatology, Seoul St. Mary’s Hospital, College of Medicine, The Catholic University of Korea, Seoul, Republic of Korea; c Opticho Inc., Pohang, Republic of Korea; d Department of Biomedical Engineering and Medical Sciences, College of Medicine, The Catholic University of Korea, Seoul, Republic of Korea

**Keywords:** Photoacoustic imaging, Ultrasound imaging, Vitiligo, Hypopigmentation, Idiopathic guttate hypomelanosis

## Abstract

Vitiligo needs early identification for proper intervention. Current adjunct diagnostic methods rely mostly on subjective visual inspection. Thus, identification of early or atypical vitiligo lesions among other hypopigmentation disorders may pose challenges. To overcome this, we investigate the feasibility of a three-dimensional (3D) photoacoustic (PA) and ultrasound (US) imaging technique as a new adjuvant analytic tool providing quantitative characterization of hypopigmentation features. This cross-sectional study was conducted at Seoul St. Mary’s Hospital (Seoul, Republic of Korea) between August 2022 and January 2024. Lesions diagnosed vitiligo or IGH in locations that could safely be irradiated with laser were analyzed with 3D PA/US imaging along with the conventional diagnostic methods. A total of 53 lesions consisted of 36 vitiligo lesions and 17 IGH lesions from 39 participants with confirmed diagnosis were analyzed. The PA amplitude greatly differed between normal skin and hypopigmentation lesions, and the mean PA amplitudes of vitiligo lesions were slightly higher than that of IGH [mean (standard deviation, SD): vitiligo: 0.117 (0.043); IGH: 0.135 (0.028)]. The local SD of the PA amplitude were higher in IGH than in vitiligo lesions [vitiligo: 0.043 (0.018); IGH: 0.067 (0.017)]. The mean PA slope across the lesion boundary was significantly higher in IGH than in vitiligo [vitiligo: 0.173 (0.061); IGH: 0.342 (0.099)], whereas the PA peak depth was deeper in vitiligo than in IGH [vitiligo: 0.568 (0.262); IGH: 0.266 (0.116)]. Unlike conventional qualitative methods, 3D PA/US imaging can non-invasively provide quantitative metrics which might aid in the differentiation of vitiligo from IGH lesions.

## 1 Introduction

Vitiligo is a depigmentation disorder impacting an estimated 0.5–2 % of the global population [1], [2], [3], [4], [5]. It is recognized as an autoimmune disease, linked to a combination of genetic and environmental factors [5], [6], [7]. Vitiligo may show spreading of pigment loss, and it is relatively common for the disease to relapse after stopping treatment and reported to be related with various psychiatric disorders [8], [9]. Treatment of vitiligo differs by various factors such as the size and activity of the lesions [10], [11], [12]. Ultraviolet (UV) B phototherapy and targeted phototherapy are widely practiced treatments, but it can take from 12 to 24 months for repigmentation [13], [14]. Therefore, early identification and initiation of treatment in the early stage of vitiligo is the key to its effective management.

Idiopathic guttate hypomelanosis (IGH) is characterized by multiple, well-defined, round to oval, 2–6 mm-sized hypopigmented macules [15]. Occasionally, there might be larger lesions reaching up to 2.5 cm [15]. Its pathogenesis is suggested to be influenced by various factors, including aging and sun exposure [16]. IGH typically affects individuals aged 40 and older, and its likelihood increases as people age [17], [18]. Although treatment is not necessary in most cases, various treatments have been attempted for lesions that can cause cosmetic concerns, but there is still no standardized treatment method [15], [17], [19], [20].

Distinguishing vitiligo lesions from other hypopigmented lesions in the early stage can be challenging. Generally, diagnosis is made through visual examination supported by supplemental methods and tools, considering clinical characteristics. A dermoscope can help in judging clinical features aiding in diagnosis [21], but if the features are not evident especially in the early stage, identifying accurate lesion boundary and presence of poliosis can be problematic. Photography can offer a visual aid for lesion boundary estimation as well as for monitoring the progression [22], [23], while it is heavily influenced by lighting and viewing angle. A skin biopsy can directly confirm the reduction of melanocytes and melanin. However, it is painful, invasive, and has limitations such as sampling errors and the inability to examine the entire lesion area [24]. A Wood's lamp, the most commonly used tool, allows for visualization of pigment loss over a large area by detecting fluorescence from the exposed collagen layer excited by UV light. Many studies have established the effectiveness of the Wood’s lamp [25], [26], [27], [28], [29], but it is still subjective and prone to inaccuracies of qualitative assessment of visual features.

Photoacoustic (PA) imaging is a non-ionizing biomedical imaging modality based on the conversion of optical energy to acoustic energy, referred to as the PA effect [30]. When a laser pulse irradiates a tissue, ultrasound (US) waves are induced according to the light absorption characteristics of the optical absorbers in the tissue. These waves are then detected with US transducers to form PA images via various image reconstruction algorithms [31], [32], [33], [34], [35], [36], [37]. Different from optical imaging methods, PA imaging uses the acoustic signal induced from optical excitation, which allows the detection of non-superficial targets up to centimeters deep, and thus the distribution of optical absorbers can be analyzed in three dimensions. Further, PA imaging is not affected by the surrounding light in the examination room, providing a more reliable quantification of the features in the image compared to optical methods. Because PA imaging relies on the optical absorption properties of chromophores, spectroscopic imaging can selectively visualize endogenous substances such as hemoglobin [38], melanin [39], collagen [40], or lipid [41]. Numerous studies have applied PA imaging from preclinical to clinical studies, and its performance has been continuously improved [42], [43], [44], [45], [46], [47], [48], [49], [50], [51], [52], [53], [54], [55], [56], [57].

PA imaging can be a promising modality for evaluating skin pigmentation diseases because melanin is a strong light absorber that is well detected with PA imaging. PA imaging can provide the concentration (i.e., PA amplitude) of melanin in three dimensions with sub-mm spatial resolution. PA imaging has been compared with other conventional methods to quantify melanin in the skin, and its performance was not inferior to them [58], [59]. Numerous PA imaging studies have assessed melanin distribution differences in various skin disorders, including pigment-related conditions [60], [61], [62], [63]. Representatively, PA imaging has demonstrated promising results in detecting melanoma’s boundary due to the strong melanin contrast and PA’s depth resolvability [64], [65]. Although there is a study in which PA signals were used to measure the loss of melanin in a vitiligo lesion [66], to the best of our knowledge, no clinical PA imaging studies have investigated the difference between vitiligo and other hypopigmentation lesions. There have been researches using other non-invasive optical imaging methodologies such as optical coherence tomography (OCT) or reflectance confocal microscopy (RCM) in diagnosing and evaluating vitiligo [67]. While they were able to detect cellular-level pigment loss and tissue changes with high spatial resolution, these methods focused on analyzing small areas (up to a few millimeter scale) in a lesion. PA microscopy and mesoscopy also provide high spatial resolution to clearly identify the layers of the skin and analyze the detailed structures [49]. They have been investigated in skin pigment disorders such as port-wine stain to analyze the characteristic features of pigment in larger field-of-views (FOVs) than OCT or RCM. For skin diseases with macroscopic lesions such as vitiligo, it is important to assess a whole lesion to make a more reliable diagnosis and perform proper treatments. In this sense, PA tomography has the strength that it can scan the entire lesion at once for a holistic view of melanin changes with a much larger FOV of centimeter scale.

In this study, we observed characteristic PA features of the two types of hypopigmentation, vitiligo and IGH, in patients using a clinical three-dimensional (3D) PA/US imaging system. PA images were co-registered with US images to localize the skin signals that represent the melanin distribution regardless of the skin pigment hypopigmentation. Using the difference in PA amplitudes between normal and lesion areas, we delineated the lesion area and analyzed the features quantitatively. We derived quantitative metrics from the PA images and evaluated their significances in differentiating vitiligo from IGH lesions.

## 2 Methods

### 2.1 3D clinical PA/US imaging system and imaging procedure

**Fig. 1:**
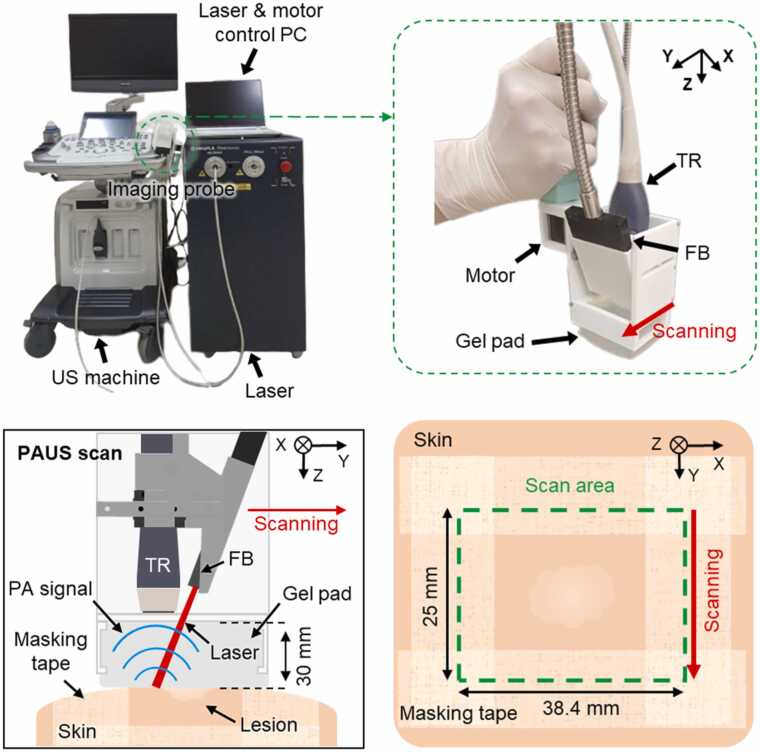
Configuration of the 3D PA/US imaging system. The photograph of the system shows the entire view of the 3D PA/US imaging system and the zoomed handheld scanner (green dotted box). The schematic in the lower half of the figure presents the scan direction and area of the handheld scanner. The FB and TR are fixed by the holder and moves together during scanning. *PA*, photoacoustic; *US*, ultrasound; *FB*, fiber bundle; *TR*, transducer.

Fig. 1 shows the 3D PA/US imaging system, which combines a programmable US imaging system (E-CUBE 12R, Alpinion Medical Systems), a tunable pulsed laser (PhotoSonus M, Ekspla), and a customized handheld scanner. The scanner consists of a 128-element linear array US transducer (L3–12, Alpinion Medical Systems) with a center frequency of 8.5 MHz and a pitch of 0.3 mm, a linear fiber optic bundle, a probe adapter, and a step motor for scanning. The handheld scanner was previously developed, based on the Scotch yoke mechanism that converts the rotational motion of the step motor into linear motion [68], [69]. The maximum scan area of the scanner is 38.4 (X) × 25.0 (Y) mm^2^, and we marked the region of interest (ROI) with clinical marking tape (Fig. 1). For each scan, 83 PA/US image pairs are obtained at an optical wavelength of 680 nm because it is predominantly absorbed by melanin among other chromophores such as hemoglobin. The laser energy emitted to skin was < 5 mJ/cm^2^, which satisfies the laser exposure limit for skin [70]. For each lesion, we perform three separate scans and selected the one with the fewest motion artifacts. The entire scanning process, including positioning and data saving, takes approximately 10 min.

### 2.2 Study design, setting, and participants

**Fig. 2:**
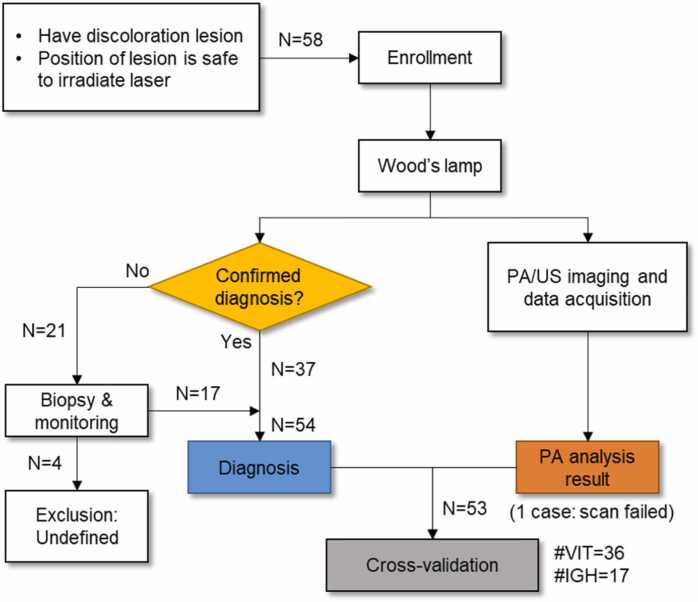
Patient selection and study work flow. *N*, the number of lesions; *PA*, photoacoustic; *US*, ultrasound; *VIT*, vitiligo.; *IGH*, idiopathic guttate hypomelanosis.

Among patients with vitiligo and IGH, those whose lesions were located in safe locations for laser irradiation were enrolled in the study. These patients were diagnosed with conventional methods, including the Wood's lamp test and skin biopsy, if needed. Lesions that were not likely to be either vitiligo or IGH or could not be diagnosed for reasons such as follow-up loss (i.e., the patient did not return to the hospital, leaving only uncertain diagnosis) were excluded from the study. Confirmed diagnoses were compared to the results of the PA/US imaging data analysis (Fig. 2). Reporting for this study was performed in accordance with the Strengthening the Reporting of Observational Studies in Epidemiology (STROBE) guidelines [71]. All the protocols were approved by the Institutional Review Board of Seoul St. Mary’s Hospital, Republic of Korea (KC17DESI0201).

### 2.3 PA image pre-processing

With acquired 3D PA/US image data, pre-processing was performed to distinguish the hypopigmented lesion and normal skin based on the difference in their PA amplitudes before quantification.

To analyze the signals solely from the epidermal and dermal skin layers, each cross-sectional PA/US image was segmented up to the skin layer depth, using the US-detected skin contour. The skin surface contour was outlined using thresholding on the US intensity and refined using pixel connectivity analysis from the US images. This procedure was applied to the PA images to remove PA signals below the dermal layer (e.g., blood vessels), leaving only PA signals from the skin surface. Because the thickness of the skin layer varies from person to person and across the parts of the body, the depth range was carefully selected to cover the PA signal with a laterally continuous pattern (corresponding to the skin contour) in each cross-sectional PA image.

**Fig. 3:**
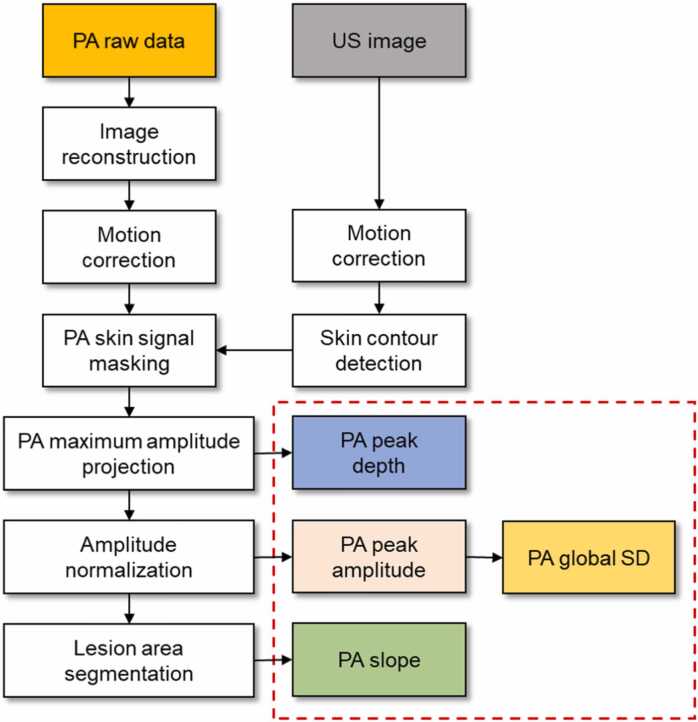
Data processing flow *US*, ultrasound; *PA*, photoacoustic; *SD*, standard deviation.

The PA signals of the lesion consisted of either the remaining melanin pigment in the epidermal layer or from the dermal layer with signals mostly comprising of hemoglobin in blood vessels. PA amplitude was relatively high in the normal skin region due to the prominent melanin distribution, but significantly weak in the hypopigmentation lesions. The 3D PA image of the skin was represented as a maximum amplitude projection (MAP) image, and the image was interpolated in the elevation direction (Y) to convert the sinusoidal step size of the scanner into a uniform step size of 0.1 mm. Likewise, the PA MAP image was also interpolated in the lateral (X) direction with the same step size (0.1 mm) in the elevation direction. A tape-marked boundary was used to limit the ROI, because the area covered with the tape made the least PA/US signal. PA MAP images showed the lesional areas with significant decreases in the PA amplitude, from which the lesion boundary was automatically segmented using Otsu’s thresholding method [72]. The images were normalized and saturated to make a clear difference between lesion and normal signals, and then blurred to prevent overfitted boundary. Next, further smoothing was performed by removing outliers based on the connectivity of pixels. Finally, the PA images were separated into two areas: lesion and normal, and corresponding boundary lines were extracted. Quantitative PA metrics were derived from the normalized PA MAP image: mean PA amplitude, local standard deviation (SD) of the PA amplitude, the PA slope across the lesion boundary and the relative PA peak depth (Fig. 3). The mean PA amplitude represents the overall amount of pigment, and the local SD of the PA amplitude was introduced to compare local pigment distribution pattern. The PA slope was calculated to observe the pigment gradient across the lesion boundary which could be related to the spreading features. The PA peak depth was measured to present the likelihood of PA peak signals being detected deeper beneath the epidermis if more pigment is lost from the epidermis.

**Fig. 4:**
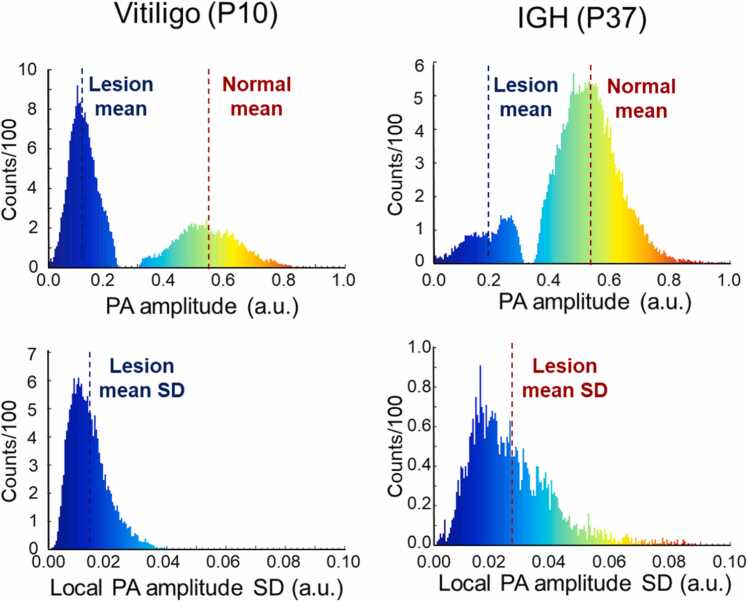
Sample histograms of the PA peak amplitude. *PA*, photoacoustic; *IGH*, idiopathic guttate hypomelanosis; *SD*, standard deviation.

**Fig. 5:**
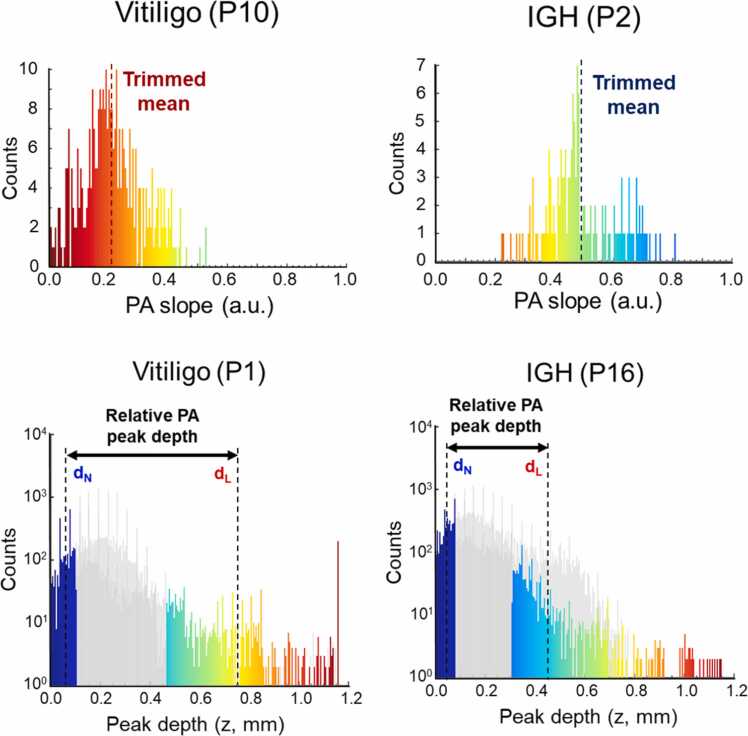
Sample histograms of the PA slope and the PA peak depth. Top rows show sample histograms of the PA slope obtained from patients 10 (vitiligo) and 2 (IGH) showing overall distribution of the PA slope tended to be lower in the vitiligo lesion than in the IGH. In bottom rows, the histograms for PA peak depths of the normal and lesion areas were plotted in superposition, where the top 15 % of lesion pixels and bottom 15 % of normal pixels were highlighted in the cases of patients 1 (vitiligo) and 16 (IGH). d_N_ is the bottom-15 % sample mean depth of the normal area and d_L_ is the top-15 % sample mean depth of the lesion area. The difference between d_L_ and d_N_ is the relative PA peak depth. *PA*, photoacoustic; *IGH*, idiopathic guttate hypomelanosis.

The mean PA amplitude was calculated as the mean of the pixel values in the lesional area of each PA MAP image, and the local SD were acquired by applying a 5 × 5 window size SD filter to each PA MAP images. To prevent the effect of outliers, SD values calculated including the boundary pixels were excluded from the mean value calculation (Fig. 4). The PA slope at each pixel on the lesion boundary was obtained as the change in the PA amplitude per unit distance across the boundary after normalizing the amplitude of the PA MAP images and then calculating their mean values to derive the mean PA signal slope across the entire boundary. First, at each point of the boundary, we selected two pixels in the PA MAP image, located at 5 pixels inward (i.e., in the lesion area) and outward (i.e., in the normal skin area) in the perpendicular direction to the boundary line. Then, the difference in the PA amplitudes between those two pixels was divided by the distance (mm) between them to represent the PA slope value at a single pixel in the boundary. Then, all the PA slope values for all the boundary pixels were sorted in order, and the mean of those values within the inter-quartile range (i.e., from 25 % to 75 %) was calculated (Fig. 5). The PA peak detection depths of each pixel in the normal and lesion areas were obtained during PA MAP processing as the location of the PA peak amplitude in the axial direction. We first created a PA peak depth projection image of the whole scan area where each pixel (X, Y) contained the depth (Z) of the peak PA signal. Using the lesion boundary information, the depth values of the normal and lesion areas were grouped and sorted in order. The bottom 15 % of the depth values from the normal skin group and the top 15 % of the depth values from the lesion group were averaged to calculate the mean PA peak depths of the normal skin (d_N_) and the lesion skin (d_L_), respectively. These two values were subtracted (d_L_ - d_N_) to get the relative PA peak detection depth for each lesion (Fig. 5). As our metrics are not ensured to follow the normal distribution, P-values for each metric were calculated using the Wilcoxon rank sum test to evaluate the significance of each metric in differentiating vitiligo and IGH.

## 3 Results

### 3.1 Information of study participants

In total, 58 skin lesions from 41 patients were collected between August 2022 and January 2024 at Seoul St. Mary’s Hospital. Five among these lesions were excluded from the PA/US image analysis: three of them were not likely to be either vitiligo or IGH, one could not be diagnosed by follow-up loss, and the other was located on an unsuitable position for imaging (philtrum). Therefore, a total of 53 lesions consisting of 36 vitiligo and 17 IGH from 39 participants with confirmed diagnoses were analyzed (Table 1; Fig. 2).

**Table 1:** Characteristics of enrolled patients and lesions.

**Characteristic**	No. (%)		
	Total	Vitiligo	IGH
**Patients**			
Total Patients, No.	39	26	13
Age at onset, median (IQR), y	60 (53−65)	57 (49.5–66.3)	62 (56−65)
Sex			
Male	11 (28.2)	8 (30.8)	3 (23.1)
Female	28 (71.8)	18 (69.2)	10 (76.9)
Fitzpatrick skin type^a^			
III	31 (79.5)	20 (76.9)	11 (84.6)
IV	8 (20.5)	6 (23.1)	2 (15.4)
**Lesions**			
Total lesions, No.	53	36	17
Location			
Face/neck	18 (34.0)	15 (41.7)	3 (17.6)
Upper extremities	27 (50.9)	19 (52.8)	8 (47.1)
Lower extremities	5 (9.4)	0	5 (29.4)
Abdomen	2 (3.8)	2 (5.6)	0
Chest	1 (1.9)	0	1 (5.9)

*IQR*, Interquartile range; *IGH*, idiopathic guttate hypomelanosis

^a^ type III, darker white skin; type IV, light brown skin.

### 3.2 PA images of the hypopigmentation lesions

**Fig. 6:**
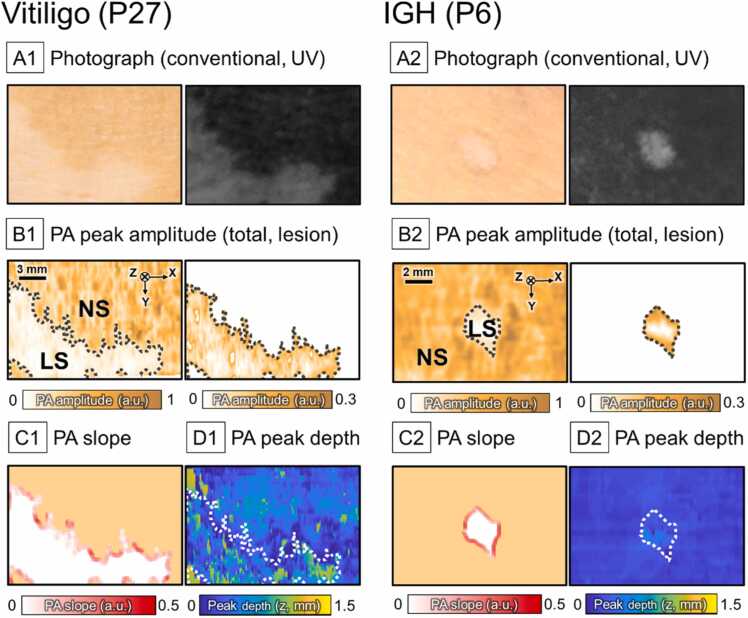
Photographs and PA images of a vitiligo lesion and an IGH lesion (P27 and P6, respectively). (A1, A2) photographs of the lesions using a conventional camera in regular room lighting (left) and a UV camera with a Wood’s lamp light (right). (B1, B2) PA peak amplitude images of lesions in total with surrounding normal tissues (left) and only lesion region (right) obtained using maximum amplitude projection. The black dotted line indicates the lesion boundary detected from the image. (C1, C2) PA slope across the lesion boundary marked onto the lesion segmentation image. The color on each square mark indicates the slope value across each point on the boundary. (D1, D2) PA peak depth image obtained from maximum amplitude projection. The white dotted line indicates the lesion boundary. *UV,* ultraviolet; *IGH*, idiopathic guttate hypomelanosis; *PA*, photoacoustic; *NS*, normal skin; *LS*, lesion skin.

Fig. 6 shows the PA images from the representative cases of vitiligo and IGH (patients 27 and 6, respectively). The photographs of the lesions were taken using a conventional camera in regular room lighting and a UV camera with a Wood’s lamp light (Fig. 6A1 and A2). The brightness and contrast of the photographs were adjusted for clarity of the lesion area. Compared to the photographs, the lesion boundaries were clearly distinguished from the PA MAP images (Fig. 6B1 and B2, left) due to the great difference in the PA amplitude between normal and hypopigmentation lesions. After segmenting the lesional area, the PA amplitude distribution was further analyzed to differentiate vitiligo and IGH (Fig. 6B1 and B2, right). The vitiligo lesion boundary showed varying levels of PA slope whereas IGH had high PA slope values (Fig. 6C1 and C2). Higher PA peak depth values were observed in the vitiligo lesion than in the IGH lesion (Fig. 6D1 and D2).

### 3.3 Statistical analysis of the quantitative PA metrics

**Fig. 7:**
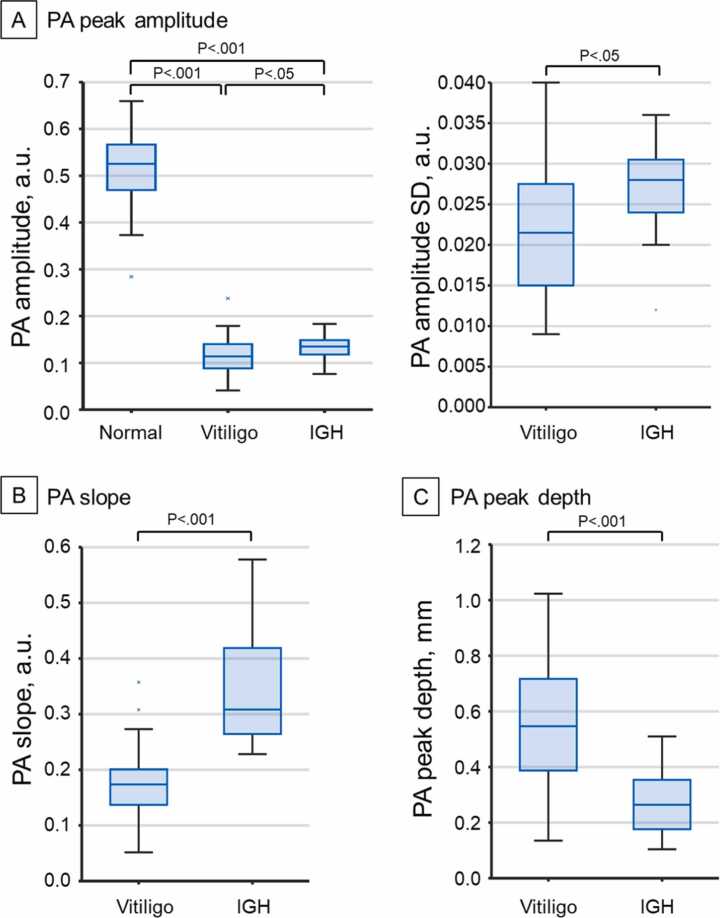
Quantitative PA analysis results. Box plots and Wilcoxon rank sum test results are shown on the top with respect to the PA analysis metrics. (A) PA peak amplitude in normal and lesions (left) and the local SD of PA amplitude in the lesion area (right). (B) PA slope across the lesion boundary. (C) PA peak detection depth. *PA*, photoacoustic; *IGH*, idiopathic guttate hypomelanosis; *SD*, standard deviation.

Fig. 7 shows the box plots of the mean PA amplitude, the local SD of the PA amplitude, the mean PA slope, and the PA peak depth. The values of both the vitiligo and IGH lesions were significantly lower than those of normal skin (Wilcoxon rank sum test, P < .001), showing that lesions can be clearly detected based on their PA amplitude. All PA metrics show statistically significant differences between vitiligo and IGH (Wilcoxon rank sum test, P < .05 for PA amplitude and the local SD of the PA amplitude and P < .001 for the mean PA slope and the PA peak depth). The mean PA amplitudes in IGH lesions were slightly higher than those in vitiligo lesions [mean (SD): vitiligo: 0.117 (0.043); IGH: 0.135 (0.028)], relevant to the different levels of pigmentation loss. Additionally, the local SD was higher in IGH than in vitiligo [vitiligo: 0.043 (0.018); IGH: 0.067 (0.017)]. The mean PA slope in IGH was significantly higher than that in vitiligo [vitiligo: 0.173 (0.061); IGH: 0.342 (0.099)], and the vitiligo lesions represented significantly greater PA peak depth values than IGH lesions [vitiligo: 0.568 (0.262); IGH: 0.266 (0.116)].

## 4 Discussion

In this study, we analyzed 3D PA/US images to quantify the features of two hypopigmented skin lesions, specifically vitiligo and IGH. Intuitively, PA amplitude could be considered as a differentiating feature if vitiligo and IGH lesions contain meaningfully different amounts of leftover melanin pigment. Both vitiligo and IGH exhibited significant decreases in PA amplitude compared to normal skin tissue. However, the mean PA amplitudes were not dramatically different between vitiligo and IGH groups. As an additional approach, we investigated the local SD of the PA amplitude to compare the heterogeneity of the melanin distribution. The mean PA local SD was significantly higher in IGH than in vitiligo. While the previous metrics quantified the pigment distribution within the lesion, the PA slope metric sought to characterize the rate of pigmentation change across the lesion boundary. The vitiligo appeared to have a more gradual mean PA slope across the lesion boundary than observed in IGH. Furthermore, the PA peak detection depth metric was presented to indicate the extent of pigment loss that ends up revealing the PA signals underneath the epidermis layer which normally melanin is distributed. Compared to IGH lesions, the PA signals from vitiligo lesions were detected at greater depths. These results showed that PA imaging quantitatively characterized the skin lesions, whereas most conventional diagnostic procedures are qualitative.

**Fig. 8:**
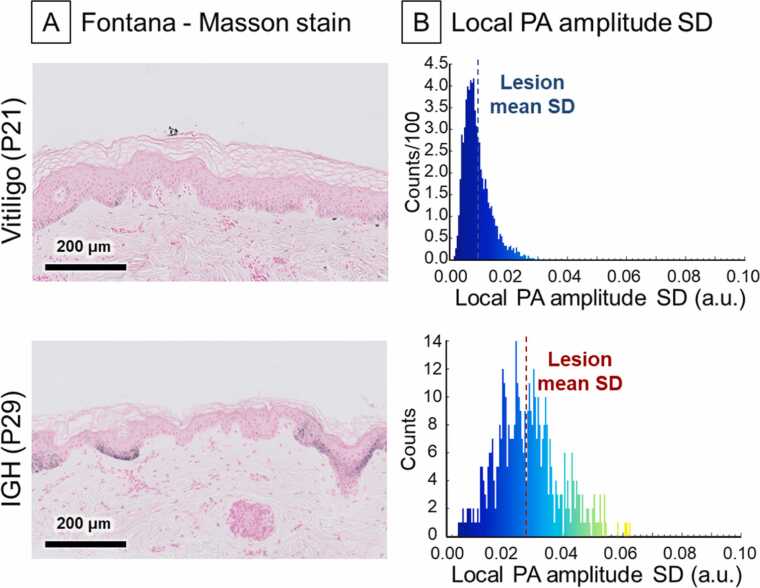
Histology images and histograms of local PA amplitude SD. A, histology images of a vitiligo and an IGH case (patients 21 and 29, respectively). In the vitiligo case, the melanin pigment (black dots) has been almost removed while some focally remained melanin group exists in IGH. B, corresponding histograms of local PA amplitude SD. The vitiligo lesion with small and relatively even reduction in pigment has a smaller SD value, while IGH has a larger SD value due to the remaining focal pigmentation. *PA*, photoacoustic; *SD*, standard deviation; *IGH*, idiopathic guttate hypomelanosis.

We initially hypothesized that vitiligo lesions would exhibit significantly lower PA amplitude levels than IGH lesions, but the results did not reveal such a great statistical significance as expected. It might be attributed to the fact that vitiligo can also retain pigment depending on the severity and activity of the condition. Note that this study performed the PA imaging at the time of the patients’ initial visits to the hospital, but the PA analysis results were compared with the final diagnoses that were confirmed later. Therefore, at the time of the PA image acquisition, the lesions could have been at relatively early stages which may not exhibit significant reduction in melanin yet. The high proportion of first-time patients (94.9 %, 37 out of 39 patients) could also contribute to the probability of early-stage hypopigmentation. In addition, PA signals from dermal microvasculature could have influenced our PA amplitude measures because they might not have been fully removed during US image-based skin contouring. Instead, we analyzed the local SD of the PA amplitude, assuming that there could be differences in the distribution pattern of retained pigment in the lesion area. IGH was suspected to have more irregular melanin decrease pattern than vitiligo because it may have characteristic focal pigmentations, previously reported as “skip areas” [73], [74]. Our measures on the local SD of the PA amplitude tended to be higher in IGH than vitiligo, which might be due to the focal pigmentation. It is still controversial, because Joshi reported that the “skip areas” were observed in almost 80 % of 55 IGH biopsies [73] while Arbache found them in only 25 % (5 cases) of 20 IGH biopsy cases [74]. Therefore, while this could be a feature of IGH that is not seen in vitiligo, we cannot guarantee that it was mostly present in our IGH cases. It also has been reported previously that these "skip areas" are not in any fixed form and can vary in size. For an IGH lesion with multifocal hypopigmentation in this study, biopsy revealed areas of the retained melanin distribution within the lesion with sizes ranging from 100 μm to several times larger than that (Fig. 8). The mean PA signal slope across the lesion boundary might indicate the spreading characteristics and the pigment status of the hypopigmentation lesions. Our result uncovered that IGH lesions had steeper PA slopes at the lesion boundaries than vitiligo lesions. This might stem from the hyperkeratosis of the stratum corneum and a more clearly defined lesional margin which could be seen in IGH, while vitiligo may retain partial melanocyte presence along their borders depending on the state and activity. The relative PA peak detection depth was evaluated assuming that the absence of melanin at the epidermal layer would reveal the PA signal from the dermal layer. In normal skin areas, the PA signals from melanin pigment in the epidermal layer were stronger than the PA signals from the dermal layer. In vitiligo lesions, due to the absence of melanin, the peak PA signal tended to be detected beneath the epidermal layer. In IGH lesions, on the other hand, the peak PA signals were expected to be less frequently observed from the dermal layer due to the remaining melanin in the epidermal layer. As the result, the relative PA peak detection depth presented to be deeper for vitiligo lesions than IGH lesions, which might be related to the difference in the amount of leftover melanin as expected. Still, differences in epidermal layer thickness across body parts were not considered, and the spatial resolution of the system needs to be improved for better precision of the measurement. While our analysis of PA metrics seemed relevant with the medical hypotheses above, further research is needed to understand the true mechanisms behind these findings.

This study is limited by the spatial resolution of the imaging probe, making it difficult to delineate the epidermal/dermal skin layers in detail. In this study, we reconstructed the images with the pixel size of 100 μm but the actual achievable resolution was several times greater than that, so the system might not be able to precisely describe the microscopic changes. To cover the whole boundary of the lesion and analyze it more precisely, a higher-frequency US transducer array with a wide lateral footprint could be used to provide both higher axial resolution as well as a large FOV. Further, spectral unmixing of multi-wavelength PA images would help identify melanin from other chromophores such as hemoglobin. Additionally, multispectral PA imaging could aid the differentiation of melanin from underlying vasculature (i.e., hemoglobin). Moreover, with sufficiently large and balanced sample size, making meaningful comparisons between subpopulations such as vitiligo groups with different activity levels might be possible. In this study, all the patients were Korean, and there was no significant variation in the skin color in terms of the Fitzpatrick grade. Future studies with a larger population of patients with various skin tones may provide more generalized and scalable results.

This study demonstrates the capability of PA imaging to non-invasively analyze and quantify the pigment status of skin possibly used in making diagnosis. While additional research is necessary, it is suggested that PA imaging could be considered for monitoring vitiligo by analyzing PA metrics might representing direct or indirect clinical features related to vitiligo activity and prognosis. This approach might further offer a supportive role in reducing dependence on patient recollection and possibly lessening the frequency of unnecessary diagnostic procedures and extended observation.

## 5 Conclusion

In this study, we investigated the usefulness of 3D PA/US imaging in differentially analyzing the features of vitiligo and IGH lesions. The low melanin content in both lesions was represented as a significant decrease in the PA amplitude compared to normal skin, based on which the lesion boundaries were clearly delineated. We also observed differences in the PA images regarding the pigment distribution inside the lesion area and across the lesion boundary. Accordingly, the lesions were characterized in terms of four PA feature metrics: the mean PA amplitude, the local SD of the PA amplitude, the mean PA slope, and the PA peak depth. All these PA metrics showed statistically significant differences between vitiligo and IGH (P < .05), and these results might be correlated with the relevant clinical features. This work suggests that 3D PA/US imaging can potentially have a supportive role in triaging the hypopigmented skin lesions quantitatively.

## Ethical approval

Reviewed and approved by the Institutional Review Board of Seoul St. Mary’s Hospital (KC17DESI0201).

## Ethics statement

The patients in this manuscript have given written informed consent to the publication of their case details.

## CRediT authorship contribution statement

**Esther Kim:** Validation, Methodology. **Junho Ahn:** Investigation, Formal analysis. **Ju Hee Han:** Writing – review & editing, Writing – original draft, Validation, Resources, Methodology, Investigation, Data curation. **Minseong Kim:** Writing – review & editing, Writing – original draft, Visualization, Software, Methodology, Investigation, Formal analysis. **Wonseok Choi:** Writing – review & editing, Writing – original draft, Supervision, Project administration, Methodology, Conceptualization. **Chulhong Kim:** Writing – review & editing, Supervision, Project administration, Funding acquisition, Conceptualization. **Ji Hyun Lee:** Writing – review & editing, Supervision, Resources, Project administration, Funding acquisition, Conceptualization. **Chul Hwan Bang:** Validation, Methodology.

## Declaration of Competing Interest

M. Kim, J. H. Han, J. Ahn, E. Kim, C. H. Bang, J. H. Lee, and W. Choi declare no competing interests. C. Kim has financial interests in OPTICHO, which, however, did not support this work.

## Data Availability

Data will be made available on request.
